# Vitexin Protects Against Scopolamine-Induced Cognitive Impairment by Preserving Synaptic Integrity and Modulating Nrf2/HO-1 and NF-κB Signaling Pathways

**DOI:** 10.1007/s12035-026-05947-0

**Published:** 2026-06-25

**Authors:** Caner Yildirim, Sena Cevik, Ramazan Bal, Davut Sinan Kaplan, Senay Gorucu Yilmaz, Hasan Ulusal, Saadet Bekerecioglu

**Affiliations:** 1 Department of Physiology, Faculty of Medicine, Gaziantep University, Gaziantep, Turkey; 2 Faculty of Health Sciences, Department of Nutrition and Dietetics, Gaziantep University, Gaziantep, Turkey; 3 Department of Medical Biochemistry, Gaziantep Islamic University of Science and Technology, Gaziantep, Turkey

**Keywords:** Vitexin, Scopolamine, Cognitive impairment, Oxidative stress, Cholinergic dysfunction, Synaptic plasticity

## Abstract

Alzheimer’s disease is characterized by progressive cognitive decline driven by oxidative stress, neuroinflammation, and synaptic dysfunction. This study investigated the neuroprotective effects of vitexin in a scopolamine (Sco)–induced rat model of cognitive impairment. Forty-two male Wistar rats were randomly assigned to six groups (*n* = 7 per group): saline, Sco (2 mg/kg/day, i.p.), Sco + vitexin (30 mg/kg/day, oral), Sco + donepezil (1.5 mg/kg/day, i.p.), vitexin alone, and donepezil alone. All treatments were administered for 14 consecutive days. Behavioral assessments using the morris water maze and elevated plus maze revealed that Sco significantly impaired spatial learning and memory while increasing anxiety-like behaviors. Vitexin treatment markedly improved these deficits, with efficacy comparable to donepezil. Biochemically, Sco elevated acetylcholinesterase activity, lipid peroxidation, and oxidative/nitrosative stress markers (TOS, OSI, MDA, Peroxynitrite, NO, and NOS) while decreasing total antioxidant status (TAS). Vitexin reversed these changes. Western blot and immunofluorescence analyses demonstrated that Sco reduced hippocampal BDNF, GDNF, PSD95, and synaptophysin levels and increased GFAP, IL-6, TNF-α, NF-κB p65, and COX-2 expression. Vitexin restored neurotrophic and synaptic proteins, suppressed astrocyte activation and inflammatory signaling, and activated the Nrf2/HO-1 pathway. These findings were further supported by qRT-PCR analysis of BDNF, GDNF, GPX4, and NF-κB. In conclusion, vitexin exerts significant neuroprotective and synaptoprotective effects against Sco-induced cognitive impairment by simultaneously restoring redox balance, suppressing neuroinflammation, and preserving synaptic integrity. These results position vitexin as a promising therapeutic candidate for neurodegenerative disorders, including Alzheimer’s disease.

**Supplementary Information:**

The online version contains supplementary material available at 10.1007/s12035-026-05947-0.

## Introduction

Alzheimer’s disease (AD) is a progressive neurodegenerative disorder characterized by the accumulation of amyloid beta (Aβ) and hyperphosphorylated Tau, molecular alterations that disrupt the central cholinergic system and drive irreversible cognitive impairment [1]. Among the brain regions affected, the hippocampus is particularly susceptible due to its essential role in the formation, consolidation, and retrieval of episodic and contextual memories in both humans and animals [2]. Neuronal loss or dysfunction within this area results in marked impairments in learning capacity and the retention of newly acquired information. Moreover, hippocampus-dependent cognitive functions are highly sensitive to oxidative stress, neuroinflammation, and synaptic deterioration, all of which contribute substantially to the progression of memory deficits. Cholinergic neurotransmission, which supports learning, memory, and neuroplasticity, is also markedly reduced in the later stages of AD, further exacerbating cognitive decline [3].

Scopolamine (Sco), a nonselective muscarinic acetylcholine receptor antagonist, disrupts cholinergic neurotransmission, which leads to impairments in learning, memory consolidation, cognitive flexibility and anxiety-like behaviors [4–7]. The Sco-induced model accurately replicates various pathological characteristics of AD, such as cholinergic dysfunction, cognitive impairment, oxidative stress, neuroinflammation, and synaptic degeneration. Sco exposure increases acetylcholinesterase (AChE) activity and triggers oxidative and nitrosative stress, as indicated by reduced antioxidant enzyme activity and increased lipid peroxidation [8–10]. Additionally, neuroinflammatory responses are initiated, marked by increased levels of nuclear factor kappa B (NF-κB) and tumor necrosis factor-α (TNF-α) in the hippocampus [11]. The pathological changes are associated with diminished expression of neurotrophic factors—including brain-derived neurotrophic factor (BDNF)—and synaptic dysfunction, which is indicated by lower levels of synaptophysin (SPN) and postsynaptic density protein 95 (PSD95), both critical for preserving synaptic integrity and plasticity [8]. Increased microglial activation, elevated production of pro-inflammatory cytokines, and heightened generation of reactive oxygen and nitrogen species (ROS/RNS) contribute to neuronal injury and cognitive decline. Current therapeutic strategies, including cholinesterase inhibitors (e.g., donepezil (Don)) and N-methyl-D-aspartate receptor antagonists, provide only symptomatic relief and are unable to deal with essential pathological processes, including oxidative stress, neuroinflammation, and synaptic dysfunction [12]. Consequently, in this neurodegenerative disease there is a critical requirement for new treatment approaches that are aimed at restoring these essential processes.

Vitexin (Vit) (*apigenin-8-C-β-D-glucopyranoside*), also known as *Mujingsu* in Chinese, is a potent bioactive flavonoid monomer widely isolated from various medicinal plants, such as *Ficus deltoidea*, bamboo, and dried hawthorn leaves (*Crataegus pinnatifida*) [13]. Research indicates that Vit has potent free radical scavenging properties and acts to preserve antioxidant enzymes, thereby efficiently eliminating oxidative cellular damage [14–16]. Vit diminishes the overproduction of reactive oxygen species (ROS) in neurological diseases by activating the nuclear factor erythroid 2–related factor 2 (Nrf2) and heme oxygenase 1 (HO-1) pathway, thus resulting in the enhanced expression and activity of essential antioxidant enzymes [17, 18]. Additionally, Vit may suppress NF-κB, TNF-α, and interleukin 6 (IL-6) signaling, which results in diminished production of pro-inflammatory mediators and a reduction in neuroinflammatory responses [19, 20]. Moreover, Vit activates Nrf2, which boosts the cellular antioxidant defense system and supports neuronal survival by upregulating BDNF expression [21]. Previous research that examined the effects of Vit on Sco-induced memory impairment in rats reported that vitexin treatment significantly improved memory recall. However, despite these findings demonstrating the neuroprotective efficacy of Vit against Sco-induced cognitive dysfunction, the underlying molecular mechanisms remain insufficiently elucidated [22]. Vit’s ability to help neurons survive, reduce inflammation, and scavenge free radicals may help protect neurons and keep synapses healthy. Elucidating the effects of Vit on specific molecular pathways could improve the understanding of its therapeutic effectiveness in cognitive disorders linked to neurodegeneration like AD. Furthermore, Vit has undergone assessment in clinical studies (NCT01647984, NCT04245761), including a phase IV research as part of a nutraceutical formulation (NCT01647984), indicating its potential for therapeutic use in neurodegenerative and cognitive diseases, including AD. Therefore, the present study aimed to investigate the neuroprotective effects of Vit against Sco-induced cognitive impairment in rats, with particular focus on the Nrf2/HO-1 antioxidant pathway, NF-κB-mediated neuroinflammation, and synaptic protein expression.

## Materials and Methods

### Experimental Animals and Housing Conditions

This research was conducted at the Gaziantep University Experimental Animal Research Center (GAUNDAM) in compliance with ethical standards and after obtaining approval from the local ethics committee (decision number 2024/73, protocol number 422). Forty-two male Wistar Albino rats, aged 8 to 10 weeks and weighing between 200 and 250 g, were utilized in this study. All procedures adhered to the standards set forth for the Care and Use of Laboratory Animals. Throughout the study, every effort was made to reduce animal distress. Moreover, all animal experiments adhere to the Animal Research: Reporting of In Vivo Experiments (ARRIVE) guidelines. During the study, all animals were housed in standard polycarbonate enclosures featuring corn cob bedding, which was cleaned daily to ensure that optimal sanitation requirements were maintained. The animals were provided with a standard rodent chow from DSA Agrifood Products Inc., Türkiye, and had ad libitum access to water. Before and throughout the experiment, the animals were kept in laboratory cages within a controlled environment featuring a 12-h light/12-h dark photoperiod and a stable temperature of 21 °C ± 2 °C, regulated by an automatic control system.

### Assignment of the Rats to Groups and Drug Administration

Animals were categorized into six groups to evaluate the impact of Vit on a cognitive impairment model induced by Sco, to compare its potential protective benefits with Donl, and to assess the effects of Vit and donepezil on healthy animals. As a result of the power analysis conducted , it was calculated that—assuming a medium effect size (*f* = 0.25)—a minimum of seven animals per group would be required to achieve 80% power at a 95% significance level of α = 0.05. The groups were classified as follows.

**Saline group**: Animals received 0.9% sterile saline once daily via both oral gavage and intraperitoneal (i.p.) injection for 14 consecutive days serving as the vehicle control.

**Sco group**: Sco was administered intraperitoneally at a dose of 2 mg/kg/day for 14 days [23, 24].

**Sco + Vit group**: Animals received Sco (2 mg/kg/day, i.p.) for 14 days and Vit (30 mg/kg/day, oral gavage) for 14 days [23–25].

**Sco + Don group**: Sco (2 mg/kg/day, i.p.) was administered for 14 days, and Don (1.5 mg/kg/day, i.p.) was administered 30 min prior to the Sco injection for 14 consecutive days [26].

**Vit group**: Vit was administered via oral gavage at a dose of 30 mg/kg/day for 14 days. This group was included to evaluate the possible positive or negative effects of Vit on healthy animals [25].

**Don group**: Don was administered intraperitoneally at a dose of 1.5 mg/kg/day for 14 days [26].

Before the experiment began, the animals were weighed to determine their body weights and were tagged with numbered ear tags for identification. Thereafter, the rats were assigned to groups using a simple randomization method by an independent researcher blinded to the study design and group allocation. After the group assignments were completed, the animals were allowed to acclimate to the new environment and to each other for a period of one week. All drug doses, routes of administration, and durations were selected based on previously published studies in the literature. Drug administrations were conducted by the same experimenter at the same time of day to reduce variability associated with handling and circadian factors.

### Behavioral Experiments

Behavioral experiments were conducted once after the disease model induction and drug treatments were completed for all groups. The experiments were performed with a single repetition in each group. The behavioral tests were designed to assess anxiety levels using the Elevated Plus Maze (EPM) test and memory impairment was tested using the Morris Water Maze (MWM) test.

### Morris Water Maze Test

This test was utilized to assess spatial learning and memory performance. The apparatus consisted of a circular pool (150 cm diameter × 50 cm height) filled with water at a 24 °C ± 1 °C, made opaque with nontoxic black paint. A transparent escape platform with a diameter of 10 cm was positioned 1 cm beneath the water’s surface in the target quadrant. Each rat participated in four trials daily over a period of four consecutive days, with a maximum trial duration of 120 s and an inter-trial interval of 30 s. In each trial, a rat was released from one of four randomly selected start positions, oriented toward the wall of the pool. If the rat did not find the platform within 120 s, it was gently directed to the platform and permitted to stay there for five seconds to learn its location. In the MWM experiment, spatial learning and memory performance were assessed during both the probe and acquisition phases using various parameters, including the time spent and average distance in the target quadrant and latency to the first entry into the target quadrant or platform area. These parameters were automatically recorded using a Noldus-EthoVision XT video tracking system (Noldus Information Technology, Wageningen, The Netherlands) [27]. During the 4-day acquisition period, latency and distance traveled to access the platform were assessed as measures of learning acquisition. A consistent decrease in escape latency and distance over trials was interpreted as an indication of learning. On the fifth day (, the platform was removed, 60-s probe trial was conducted. The time spent in the target quadrant, the average distance in the target quadrant and platform location, and the latency to the first entry into the target quadrant or platform were analyzed as indicators of spatial memory retention. To reduce animal stress, all behavioral testing was conducted at a consistent time of day by the same experimenter.

### Elevated Plus Maze Test

This test was performed to evaluate the anxiety-related behavioral aspects commonly linked to Sco-induced cholinergic dysfunction. The apparatus comprised two open arms (50 cm × 10 cm) and two closed arms (50 cm × 10 cm × 40 cm), configured in a plus shape and elevated 90 cm from the floor. Each rat was positioned at the center of the maze, oriented toward an open arm, and permitted to explore for a duration of 5 min. The time spent in the open and closed arms, frequency of entries into each arm, and overall exploratory behavior were measured utilizing the EthoVision XT tracking system. A decrease in the proportion of time allocated to open arms was interpreted as an elevation in anxiety-like behavior. The EPM test was incorporated to enhance MWM data by assessing emotional reactivity, which may affect cognitive performance and indicate the overall neurobehavioral effects of Sco [28].

### Sacrifice and Sample Collection

After the completion of the behavioral studies, the animals were euthanized under ketamine (80 mg/kg) and xylazine (10 mg/kg) anesthesia (i.p.) [29]. The brains of the animals were quickly removed, and the hippocampal tissue was dissected and sectioned. The tissue samples were then transferred to pre-labeled Eppendorf tubes and stored at −80 °C in a freezer until further analysis.

### Biochemical Analysis

Blood samples for biochemical analyses were obtained from the inferior vena cava of anesthetized animals utilizing a 5-mL syringe, in accordance with established protocols for terminal blood collection in rodents. The blood samples underwent centrifugation at 4000 x *g* for 10 min at 4 °C to facilitate the serum and plasma separation. The levels of malondialdehyde (MDA)—which serves as a marker for lipid peroxidation—along with indicators of nitrosative stress (including nitric oxide synthase (NOS), nitric oxide (NO), and peroxynitrite), total oxidant/antioxidant status (TOS/TAS), and estimation of hippocampal acetylcholinesterase (AChE) activity were evaluated biochemically.

### Estimation of Hippocampal AChE Activity

AChE activity in the hippocampus was quantified utilizing rat ELISA kit (Acetylcholinesterase (AChE) Activity Assay Kit (Elabscience, catalog number: E-BC-K174-M), Wuhan, China). Briefly, AChE catalyzes the hydrolysis of acetylcholine to produce choline, which then combines with dithio p-nitrobenzoic acid (DTNB) to yield 5-thio-2-nitrobenzoic acid (TNB). In summary, 20 µL of tissue homogenate/serum sample was added to a microplate, followed by 170 µL of chromatographic study solution and 10 µL of substrate. Absorbance measurements were performed using a Multiskan microplate reader (Thermo Scientific, Singapore) at 412 nm, the wavelength at which TNB exhibits a distinct absorption peak at 30 and 330 s. AChE activity was determined by measuring the rate of increase in absorbance at 412 nm. One unit of enzymatic activity is defined as the amount that catalyzes the production of 1 nmol TNB by 1 mg of protein per minute. AChE results was expressed as U/mg protein.

### Total Oxidant–Antioxidant Status

The total antioxidant status (TAS) (Rel Assay Diagnostics, Türkiye) level was measured using a fully automated method. This approach involves the oxidation of reduced 2,2′-azino-bis(3-ethylbenzothiazoline-6-sulfonate) (ABTS) molecules by hydrogen peroxide (H_2_O_2_) in an acidic environment, yielding a dark green solution. Upon the addition of the 18 µL of tissue homogenate/serum sample, the antioxidants present neutralize the ABTS radicals in proportion to their concentration, thus resulting in the decolorization of the solution. The change in color intensity, which is indicative of the sample’s antioxidant capacity, was spectrophotometrically quantified at 660 nm using a Multiskan microplate reader (Thermo Scientific, Singapore). The reduction in absorbance at this wavelength is directly correlated with the TAS level [30]. The total oxidant status (TOS) (Rel Assay Diagnostics, Türkiye) level was measured using a fully automated colorimetric method. In this test, the oxidants in the 45 µl of tissue homogenate sample oxidize the ferrous ion-*o*-dianisidine complex to ferric ion. The ferric ion subsequently forms a chromatic complex with xylenol orange in an acidic environment. The intensity of the resultant color, indicative of the total oxidant molecules in the sample, was quantified at 530 nm using a microplate reader. The absorbance recorded at this wavelength is directly proportional to the TOS concentration [31]. The ratio of TOS to TAS was used as the oxidative stress index (OSI), calculated as an arbitrary unit using the following formula:

unit using the formula;

$$OSI=TOS [\mu \mathrm{m}\mathrm{o}\mathrm{l} \mathrm{H}2\mathrm{O}2\frac{\mathrm{E}\mathrm{q}}{\mathrm{L}}]\div (TAS [\mathrm{m}\mathrm{m}\mathrm{o}\mathrm{l} \mathrm{T}\mathrm{r}\mathrm{o}\mathrm{l}\mathrm{o}\mathrm{x}\frac{\mathrm{E}\mathrm{q}}{\mathrm{L}}]\times 10.$$ [32]

### Determination of Peroxynitrite

The peroxynitrite level was measured using the method described by Van Uffelen et al. [33], according to which 5 mM of phenol dissolved in 1.990 mL of 50 mM sodium phosphate buffer (pH 7.4) was added to 10 µl of tissue homogenate. The mixture was incubated at 37 °C in the dark for 2 h, after which 15 µl of 0.1 M NaOH solution was added to the mixture. Immediately afterward, the absorbance of the samples was measured at 412 nm using a spectrophotometer, and the amount of peroxynitrite was calculated using the molar extinction coefficient (ε = 4400 M⁻¹ cm⁻¹) of the nitrated phenol product.

### Determination of Malondialdehyde

The malondialdehyde (MDA) level was determined by exploiting the fact that the color formed by the reaction of MDA with thiobarbituric acid (TBA) has maximum absorbance at 532 nm [34]. For this method, 0.8 mL of phosphate buffer, 0.025 mL of butylated hydroxytoluene (BHT), and 0.5 mL of trichloroacetic acid (TCA) were added to 0.2 mL of the tissue homogenatesample. The tubes were vortexed and then kept on ice for 2 h, then centrifuged for 15 min at 2000 × *g*. Thereafter, 1 mL of the supernatant was transferred to a separate tube, followed by the addition of 0.075 mL of 0.1 M EDTA and 0.25 mL of 1% (w/v) TBA dissolved in 0.05 N NaOH. . The tubes were then vortexed, incubated in a boiling-water bath for 15 min, and cooled to room temperature; thereafter, their absorbance was measured at 532 nm using a spectrophotometer. The MDA level was determined using the molar extinction coefficient (1.56 × 10^5^ cm-1 M-1).MDA levels were expressed as nmol/mg protein.

### Determination of Nitric Oxide

Nitric oxide levels were determined according to the method described by Cortas and Wakid [35]. Briefly, the method involved deproteinization by adding 600 µL of 0.055 M NaOH to 300 µL of sample. The deproteinized sample was then added to cadmium granules activated with H_2_SO_4_ and incubated for 1.5 h. The mixture was centrifuged, and the supernatant was collected. Griess reagent was added to the supernatant, incubated, and absorbance was measured at 545 nm using a spectrophotometer. The results were expressed as μmol/g of wet tissue.

### Nitric Oxide Synthase Activity Assay

Nitric oxide synthase activity was assessed using the method described by Durak et al. [36]. Briefly, this method is based on the diazotization of sulfanilic acid at acidic pH, which combines with *N*-(1-naphthyl)-ethylenediamine to form an aliphatic diazonium complex. In this method, 200 µL of 20 mM arginine was added to 100 µL of sample and incubated for 1 h. Then, 4 mM HCl, 20 mM sulfanilic acid, and 12.5 mM *N*-naphthylethylenediamine were added sequentially to the sample in sequence and incubated for 10 min. The absorbance of this mixture was measured at 540 nm using a spectrophotometer, and the results were calculated in U/mL.

### Gene Expression Analysis

 Gene expression analysis was performed hippocampal tissues from the experimental groups. To analyze gene expression, total RNA was reverse-transcribed into cDNA using the RT Master Mix (SOLIScript® RT cDNA synthesis Kit, Solis BioDyne, Tartu, Estonia, catalog no; 06–35−00050) (20 µL reaction: 4 µL master mix, 1 µg RNA, oligo(dT)/random hexamers, 45 °C for 20 min). The cDNA was diluted (1:5–1:10) and subjected to qPCR using the QuantiNova SYBR Green PCR Kit (Qiagen, Hilden, Germany, catalog no. 330513). , (25 µL reaction: 12.5 µL master mix, 1 µL cDNA, gene-specific primers) (Molgentek, Adana, Türkiye, synthesized primers) under standard cycling conditions (95 °C for 10 min, 40 cycles of 95 °C/15 s and 60 °C/1 min) (Table 1).

**Table 1:** The table presents the genes investigated in the study, detailing their Gene IDs, GenBank accession numbers, and corresponding primer sequences (5′→3′). BDNF, GDNF, GPX4, and NFKB were assessed as target genes, with ACTB serving as the reference gene for normalization. In addition, primer sequences were optimized to ensure specific amplification of the target genes

Gene	Gene ID*	Accession No	Primer sequence (5′→3′)
BDNF	24225	NM_012513	F: GCAAGATGAAGGCGCATAGC R: CTTATGAATCGCCAGCCAATTCTC
GDNF	25453	NM_000514	F: TGCTGCGGATTCTTTATGGG R: GTAGGCCAGTCGTTTTCAGC
GPX4	29328	NM_001039847	F: GGGCAAGACCGAAGTAAACG R: TCCAGGGAAGATTTGCACGA
NFKB	81736	NM_024528	F: TGCTGACAGTGGAGAGGATG R: CCTGTGGTTCTTTGCCTCCT
ACTB	81822	NM_001101	F: AGCCATGTACGTAGCCATCC R: TCTCAGCTGTGGTGGTGAAG

Gene expression levels were normalized to β-actin (ACTB) as the reference gene. Relative quantification was performed using the 2^(−ΔΔCt) method, where ΔCt = Ct(target) − Ct(reference) and ΔΔCt = ΔCt(sample) − ΔCt(control). Fold changes in gene expression were calculated accordingly. A no-RT control was included to confirm the absence of genomic DNA contamination.

### Immunofluorescence Staining and Confocal Imaging

At the conclusion of the experiment, animals were deeply anesthetized (ketamine 80 mg/kg + xylazine 10 mg/kg, i.p.) and transcardially perfused with 0.9% saline, followed by 4% paraformaldehyde (PFA) in 0.1 M phosphate buffer (pH 7.4). Thereafter, their brains were rapidly removed and post-fixed in 4% PFA for overnight at 4 °C. The tissues were then cryoprotected in 30% sucrose solution (in 0.1 M PBS) at 4 °C until they sank. The hippocampal region was isolated for immunohistochemical analysis. Frozen coronal sections (20 μm thick) were cut and mounted on poly-L-lysine (PLL)–coated microscope slides. To block nonspecific binding, sections were incubated for 2 h at 4 °C with goat serum diluted in the ratio 1:20 in PBS (ab7481, Abcam, Cambridge, UK). Then, the samples were incubated overnight at 4 °C with the relevant primary antibodies. Sections were rinsedthree times with PBST (10 min each) and incubated with secondary antibodies for 2 h at 4 °C. . The slides were subsequently coverslipped with a mounting medium that included DAPI (Vectashield, Vector Labs). Primary antibodiesused were as follows: PSD95 (Rabbit Polyclonal, AF5283, Affinity Biosciences, 1:250)—were used to label postsynaptic sites, a marker for postsynaptic density and synaptic plasticity. Synaptophysin (Rabbit Polyclonal, AF0257, Affinity Biosciences, 1:200) was used to label synaptic vesicles, a marker for presynaptic terminals. MAP2 (Mouse Monoclonal, 13–1500, Invitrogen, 1:250) was used to label dendrites and neuronal cell bodies, a marker for neuronal morphology and structure. GFAP (Mouse Monoclonal, 14–9892−82, Invitrogen, 1:100) was used to label astrocytes, a marker for glial cells in the brain, particularly in response to neuroinflammation. APC/CC1 (Rat Monoclonal, MA5-47695, Invitrogen, 1:200) was used to label oligodendrocyte precursor cells (OPCs) and mature oligodendrocytes, a marker for myelination and glial differentiation. The secondary antibodies utilized included Donkey anti-Rabbit Alexa Fluor 594, Donkey anti-Mouse Alexa Fluor 647, and Donkey anti-Rat Alexa Fluor 488. Fluorescently labeled sections were visualized using a Zeiss LSM 900 confocal laser scanning microscope (Carl Zeiss, Jena, Germany). Images were acquired using 5× and 40× objectives in Z-stack mode. Imaging parameters (laser power, gain, pinhole size) were kept constant across all groups. For quantitative fluorescence intensity measurements, image analysis was performed using Zen software (Zeiss) and ImageJ/Fiji. Negative controls were prepared by omitting the primary antibodies (data not shown). The exported images were processed with the Adobe Illustrator software to obtain graphics.

### Western Blot Analysis

After the completion of the behavioral studies, the animals were euthanized under anesthesia (80 mg/kg ketamine + 10 mg/kg xylazine, i.p.) [29]. Brain tissue was rapidly removed , and the hippocampus of each rat was dissected thereafter. Hippocampi were individually dissected, and tissue samples were lysed using RIPA buffer containing protease inhibitors (400 µl RIPA buffer + 8 µl Protease inhibitor cocktail/50 mg tissue). Tissue samples were mechanically homogenized using a TissueLyser II (Qiagen, Germany) with 5-mm stainless steel beads at 40 Hz for 1 min. The homogenization process was performed under cold conditions, and all samples were pre-cooled to +4 °C. Following homogenization, the samples were centrifuged at 14,000 × g for 30 min at 4 °C. The resulting supernatants were carefully collected and stored at −80 °C until further analysis. The total protein levels were spectrophotometrically measured using a μDrop plate (Thermo Scientific, Multiskan SkyHigh, Singapore). After quantification, homogenates were mixed with the Laemmli sample buffer (2X) and denatured by heating at 95 °C for 5 min using a DLAB heating block (DLAB Scientific, Beijing, China). Equal amounts (20 µg) of the total protein were loaded into each lane and subjected to electrophoresis on 12% sodium dodecyl sulfate–polyacrylamide gels (SDS-PAGE) for 30 min at 80 V, followed by 180 min at 100 V. The proteins were subsequently transferred onto PVDF membranes for a duration of 80 min at a current of 400 mA. To block nonspecific antibody binding, membranes were incubated for 1 h at room temperature in TBS-T buffer supplemented with 5% skimmed milk powder for blocking. Membranes were incubated overnight at 4 °C in the dark with primary antibodies diluted in TBS-T buffer, which contained 0.1% Tween-20 and 5% skimmed milk powder. The next day, membranes underwent washing and were incubated with suitable HRP-conjugated secondary antibodies for 2 h at +4 °C. Further, we utilized the following primary antibodies: (1) β-Actin: Rabbit polyclonal, catalog number: AF7018 (dilution 1:3000). (2) TNF-α: Rabbit polyclonal, catalog number: AF7014 (1:1000 dilution). (3) PSD95: Rabbit polyclonal, catalog number: AF5283 (dilution 1:1000). (4) Synaptophysin: Rabbit polyclonal, catalog number: AF0257 (1:1000 dilution). (5) HO-1: Rabbit polyclonal, catalog number: AF5393 (dilution 1:1000). (6) Nrf2: Rabbit polyclonal, catalog number: AF0639 (dilution 1:1000). (7) NF-kB p65: Rabbit polyclonal, catalog number: AF0874 (1:1000 dilution). (8) COX-2: Rabbit polyclonal, catalog number: AF7003 (dilution 1:1000). (9) IL-6: Rabbit polyclonal, catalog number: DF6087 (1:1000 dilution). (10) BDNF: Rabbit polyclonal, catalog number: DF6387 (1:1000 dilution). (11) GFAP: Rabbit polyclonal, catalog number: DF6040 (1:1000 dilution). (12) SIRT1: Mouse monoclonal, catalog number: BF0189 (1:1000 dilution). All antibodies were purchased from Affinity Biosciences ( Cincinnati, OH, USA). Following three washes (10 min each) in 1× TBS-T, the protein bands were visualized using Clarity™ Western ECL substrate in a chemiluminescence imaging system (Fusion FX, Vilber Lourmat, Collégien, France). Following imaging, the band intensities were assessed utilizing ImageJ software (Fiji distribution, NIH, Bethesda, MD, USA). Protein expression levels were standardized against β-actin.

### Statistical Analysis

The statistical package for the social sciences (SPSS) version 23.0 (SPSS Inc., Chicago, USA) package program was used to perform statistical analysis in this study. The Shapiro–Wilk test was performed to assess the normality of data distribution. For the normally distributed data, one-way analysis of variance (ANOVA) was utilized. Following this, the Tukey post hoc test was performed to establish which group the differences stemmed from. The descriptive statistics for numerical variables were expressed as group mean ± standard deviation. Significance was considered at *p* < 0.05 0 . For the Morris water maze acquisition phase, two-way repeated measures ANOVA was used to analyze the effects of group and day, followed by Bonferroni post hoc test. Finally, the graphics were drawn using GraphPad Prism software and Adobe illustrator.

## Results

### Behavioral Experiments

#### Results of the Morris Water Maze Test

**Fig. 1:**
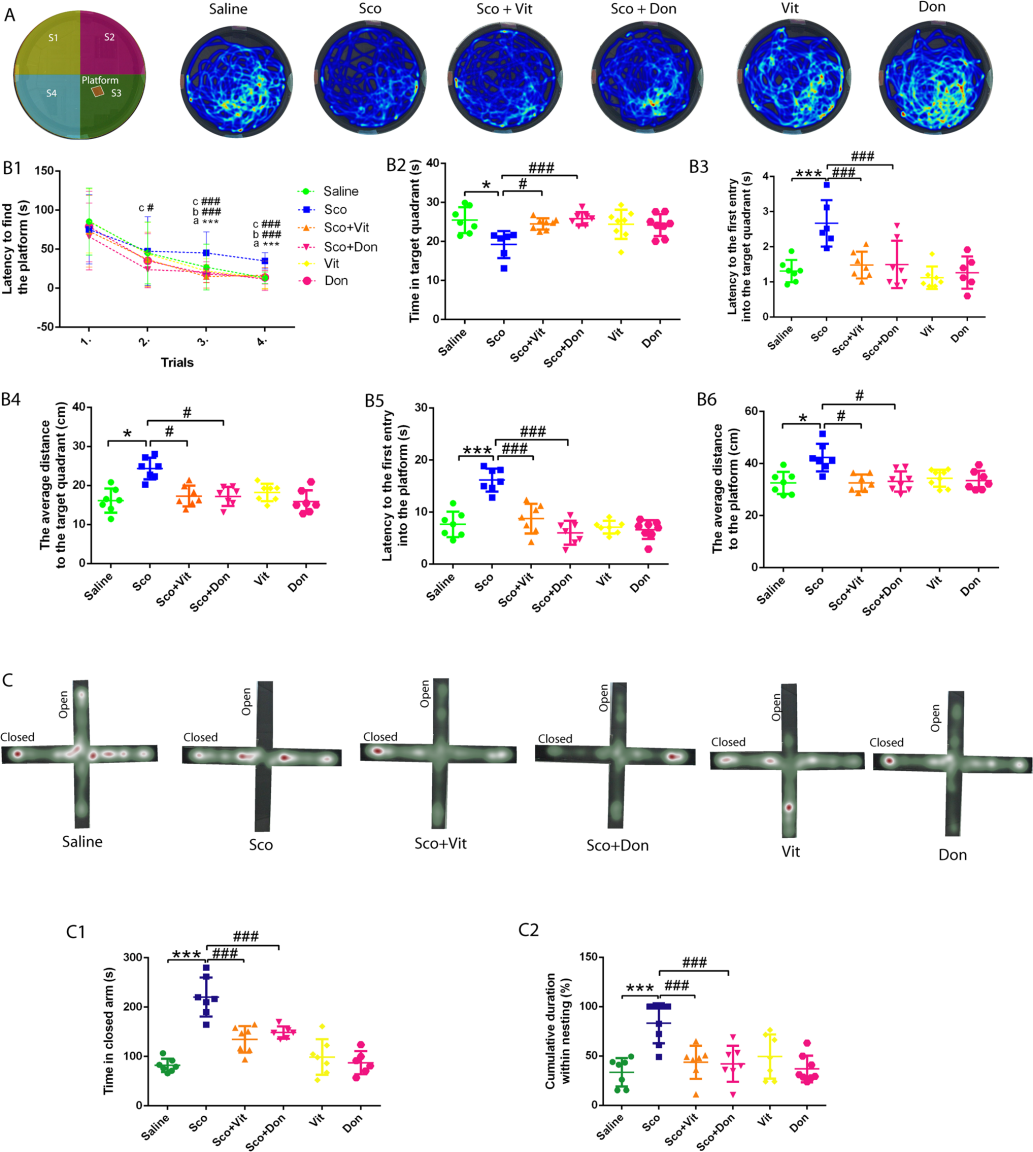
Effects of Vit and Don on cognitive deficits induced by Sco in rats, assessed through the MWM test. (A) Schematic representation of the MWM, illustrating the platform situated in quadrant S3, which was identified as the target quadrant. (A1) Representative heatmaps depict the swimming trajectories of each group during the probe trial. Warmer colors, specifically red to yellow, indicate regions of increased occupancy. (B1) Learning curves illustrate the mean first latency (time taken to find the platform) over four training trials for each group. (B2) Duration in the target quadrant during the probe test. (B3) Latency to the first entry into the target quadrant. (B4) Latency to the first entry at the platform. (B5) Mean distance to the center of the target quadrant. B6: Average distance to the platform during the probe trial. (C) Representative movement trajectories of rats from each experimental group during the five-minute EPM test. (C1) Quantitative analysis of the proportion of time allocated to the closed arms, indicative of anxiety-related behavior. (C2) Cumulative percentage of time spent in the nesting behavior throughout the testing period. Statistical significance: *p < 0.05 and ***p < 0.001 vs. saline; #*p* < 0.05 and ###*p* < 0.001 vs. Sco.

The administration of Sco caused notable deficits in spatial learning and memory. During the training phase, significant impairments in learning and memory were observed on the 3rd and 4th days in Sco-exposed rats. Specifically, Sco-exposed rats exhibited a significantly prolonged first latency to find the platform compared to the saline group; however, the administration of Vit (Sco + Vit) or Don (Sco + Don) markedly decreased latencies on the 3rd day (*F*(5, 179) = 6.641, *p* < 0.001) and the 4th day (*F*(5, 176) = 7.890, *p* < 0.001). In the probing experiment, Sco animals displayed a diminished duration in the target quadrant (*F*(5, 38) = 4.365, *p* = 0.003) and exhibited extended latencies to first entry the target quadrant (*F*(5, 39) = 7.025, *p* < 0.001) and reach the platform (*F*(5, 33) = 17.070, *p* < 0.001), as well as increased average distances to both the target quadrant (*F*(5, 28) = 4.500, *p* = 0.004) and the platform (*F*(5, 28) = 4.156, *p* = 0.006). Treatment with Vit and Don markedly ameliorated the inadequacies induced by Sco, thus increasing time allocated to the target quadrant while reducing latencies and distances relative to the Sco group (*p* < 0.05–0.001). The Vit and Don groups without Sco exposure had comparable performance to that of the saline group, thereby indicating an absence of adverse cognitive effects in healthy subjects (Fig. 1A and B).

#### Results of the Elevated Plus Maze Test

The elevated plus maze (EPM) test results indicated that Sco administration significantly induced anxiety-like behaviors in rats. The representative heatmaps and quantitative analyses indicated that the Sco group spent significantly more time in the closed arms as compared to the saline group (*F*(5, 32) = 22.869,* p* < 0.001), thereby suggesting increased anxiety levels. Moreover, the cumulative duration of immobility (freezing behavior) was significantly greater in the Sco group compared to the saline group (*F*(5, 36) = 7.579, *p* < 0.001), thus indicating increased anxiety and diminished exploratory behavior. Treatment with Vit or Don significantly attenuated anxiety-like behaviors. Both treatments reduced the time spent in the closed arms and overall freezing duration compared to the Sco group. In contrast, the Vit and Don groups without Sco exposure displayed behavioral profiles comparable to the saline group, indicating that these treatments alone did not induce anxiety-like effects. Overall, these findings demonstrate that Vit and Don effectively mitigate scopolamine-induced anxiety-like behaviors in rats (Fig. 1C).

#### Results of the Biochemical Analysis

##### Evaluation of Acetylcholinesterase Activity, Oxidative–Nitrosative Stress, and Lipid Peroxidation

Administration of Sco resulted in a significant reduction in the TAS and an increase in the TOS, oxidative stress index (OSI), malondialdehyde (MDA), peroxynitrite, nitric oxide (NO) levels, and nitric oxide synthase (NOS) activity as compared to the saline group (*p* < 0.001, *p* < 0.01). Treatment with Vit or Don significantly restored TAS and decreased TOS, OSI, MDA, peroxynitrite, NO, and NOS activity (*p* < 0.001, *p* < 0.01, *p* < 0.05) vs. Sco), thus demonstrating a substantial reduction in oxidative and nitrosative stress. Vit and Don alone did not significantly affected these parameters, thereby indicating no negative influence in healthy conditions. In addition, the activity of the hippocampal acetylcholinesterase (AChE) was significantly increased in the Sco group (*p* < 0.01) compared to the saline group. Both Vit and Don treatments restored AChE activity to levels comparable to control levels (*p* < 0.05 vs. Sco), thus demonstrating the effective reversal of Sco-induced cholinergic dysfunction (Table 2).

**Table 2:** Tissue activity of AChE, levels of oxidative stress markers, antioxidant capacity indicators, and nitric oxide-related parameters. Statistical significance: *p < 0.05, **p < 0.01, ***p < 0.001 vs. saline; #*p* < 0.05, ##*p* < 0.01, ###*p* < 0.001 vs. Sco

Groups	Saline	Sco	Sco + Vit	Sco + Don	Vit	Don	*p*
AChE activity (U/mg protein)	14.3 ± 3.6	23.8 ± 6.2**	15.5 ± 3.7^**#**^	14.7 ± 2.8^**#**^	15.6 ± 2.3	14.9 ± 1.8	(*F*(5, 29) = 4.478, *p* = 0.004)
TAS (µmol Trolox eq/l)	1.75 ± 0.1	1.31 ± 0.1**	1.66 ± 0.3^**#**^	1.74 ± 0.1^**##**^	1.78 ± 0.1	1.63 ± 0.2	(*F*(5, 30) = 4.681, *p* = 0.003)
TOS (µmol H2O2 eq/l)	10.5 ± 1.7	15.5 ± 1.5***	11.9 ± 0.5^**#**^	12.3 ± 1.1^**##**^	11.8 ± 1.9	11.6 ± 1.3	(*F*(5, 30) = 8.182, *p* < 0.001)
OSI	0.60 ± 0.1	1.18 ± 0.1***	0.74 ± 0.1^**###**^	0.71 ± 0.1^**###**^	0.71 ± 0.1	0.66 ± 0.1	(*F*(5, 30) = 16.315, *p* < 0.001)
MDA (nmol/ml)	2.2 ± 0.5	4.4 ± 1.6***	2.7 ± 0.9^**#**^	2.5 ± 0.3^**#**^	2.7 ± 0.9	2.8 ± 0.8	(*F*(5, 42) = 5.335, *p* < 0.001)
Peroxynitrite (μmol/l)	11.7 ± 1.0	14.1 ± 1.1***	12.0 ± 0.8^**#**^	12.5 ± 1.0^**#**^	12.3 ± 0.8	12.4 ± 1.1	(*F*(5, 42) = 5.348, *p* < 0.001)
NO (μmol/l)	12.4 ± 0.6	18.8 ± 2.4***	14.5 ± 2.4^**#**^	14.6 ± 1.2^**#**^	15.0 ± 6.4	14.2 ± 2.2	(*F*(5, 42) = 9.704, *p* < 0.001)
NOS (U/ml)	15.1 ± 5.4	32.6 ± 9.6***	20.3 ± 6.2^**#**^	20.2 ± 4.5^**#**^	23.8 ± 8.6	19.4 ± 8.7	(*F*(5, 42) = 4.966, *p* = 0.001)

##### Analysis of Gene Expression

**Fig. 2:**
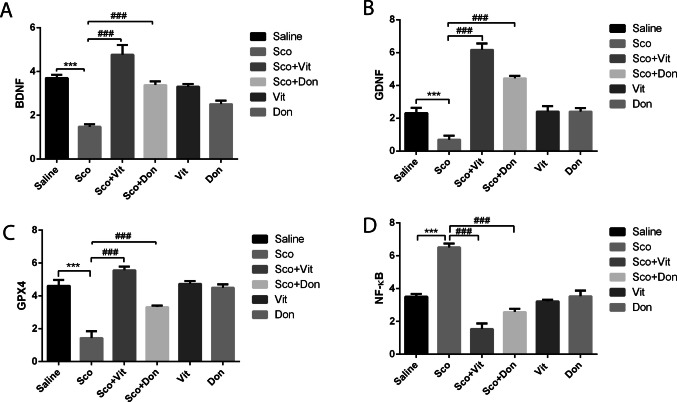
Levels of BDNF, GDNF, GPx4, and NF-κB in the hippocampus across experimental groups. (**A)** Concentrations of BDNF were assessed in the hippocampal tissues of the groups. (**B)** Levels of GDNF in the corresponding experimental groups (**C)** GPx4 levels indicate antioxidant defense capacity and cellular redox balance (**D)** NF-κB levels are a crucial indicator of neuroinflammatory processes. Statistical significance was observed at ***p < 0.001 when compared to the Saline group; additionally, ###*p* < 0.001 was considered significant when compared to the Sco group.

Exposure to Sco significantly reduced the expression of brain-derived neurotrophic factor (BDNF) and glial cell line-derived neurotrophic factor (GDNF) in comparison to these expressions in the saline group (*p* < 0.001), thus suggesting compromised neurotrophic support. Both the Sco + Vit and Sco + Don groups exhibited significantly increased expression of BDNF and GDNF when compared to these expressions in the Sco group (*p* < 0.001), thus indicating that Vit and Don effectively restore neurotrophic signaling in the hippocampus. Further, glutathione peroxidase 4 (GPx4)—an essential enzyme for cellular protection against lipid peroxidation— was significantly decreased in the Sco group, thus suggesting heightened oxidative stress. Treatment with Vit or Don significantly increased GPx4 expression levels, thereby indicating their antioxidant-enhancing effects. The expression of nuclear factor kappa B (NF-κB), a key transcription factor in inflammatory signaling, was significantly elevated in the Sco group (*p* < 0.001). Co-treatment with Vit or Don significantly reduced NF-κB expression (*p* < 0.001), thus indicating a suppression of Sco-induced neuroinflammation (Fig. 2). The gene expression results align with the behavioral, biochemical, and immunofluorescence findings, which collectively support the neuroprotective role of Vit through the modulation of oxidative stress, inflammation, and neurotrophic pathways.

##### Findings of Immunofluorescence Staining

**Fig. 3:**
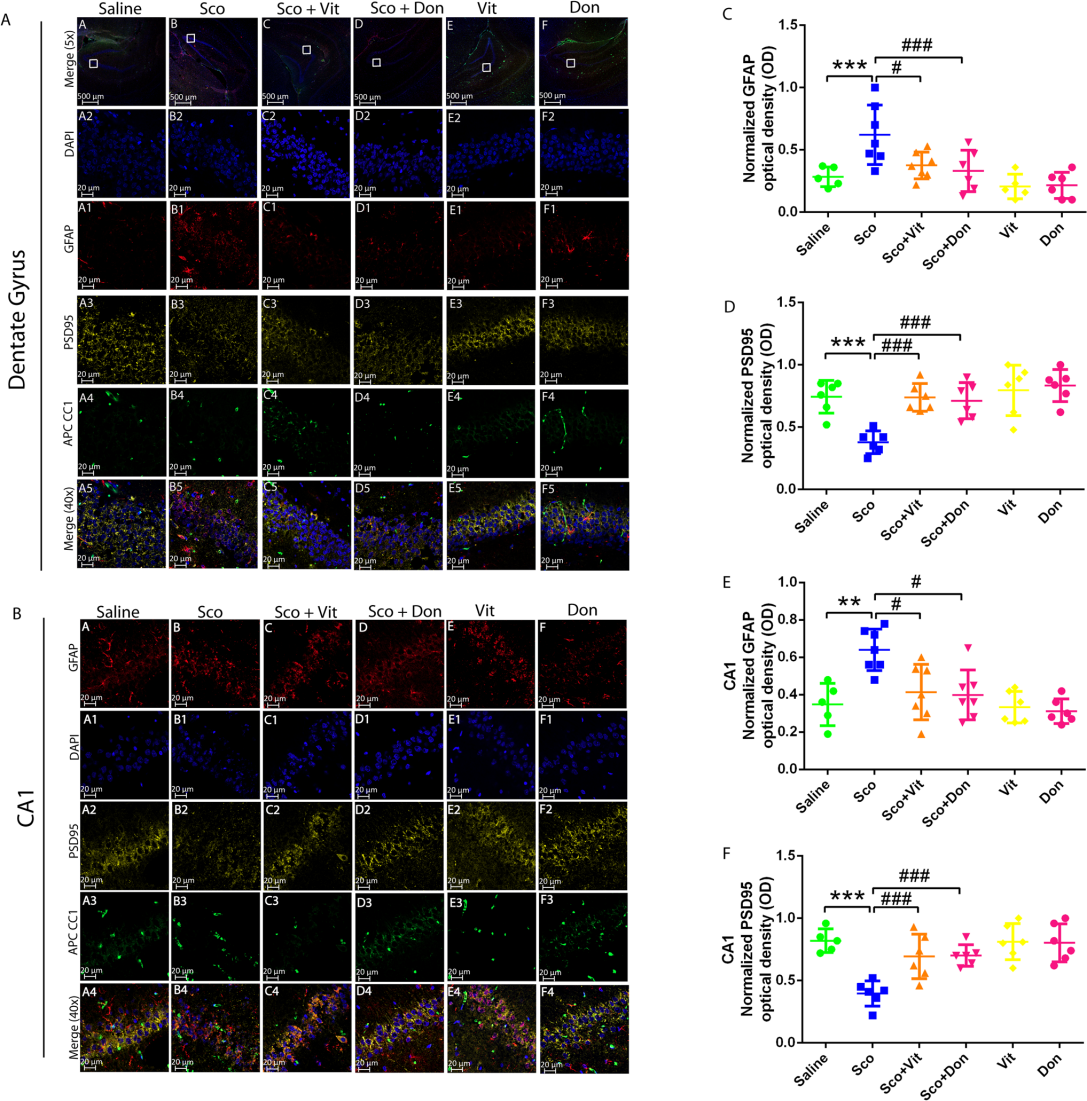
Confocal immunofluorescence images of the hippocampal dentate gyrus (**A**–**D**) and CA1 (**B**, **E**, **F**) regions illustrating GFAP (red; astrocytes), PSD95 (yellow; postsynaptic marker), APC/CC1 (green; oligodendrocytes), and DAPI (blue; nuclei). Low-magnification merged images of the dentate gyrus (**A**, top row) demonstrate the overall distribution of markers; high-magnification panels (**A** and **B**, bottom rows) present individual channels and their merged forms. Sco elevated GFAP immunoreactivity and diminished PSD95 signal, thus indicating reactive astrogliosis and suggesting synaptic impairment, respectively, in both regions. Treatment with Vit (Sco + Vit) or Don (Sco + Don) reduced GFAP elevation and maintained PSD95 levels compared to Sco alone. No alterations were noted in CC1 + oligodendrocytes or DAPI staining, thereby suggesting targeted effects on astrocytes and synapses while preserving neuronal and oligodendrocytic integrity. The quantitative optical density of GFAP and PSD95 is presented in panels **C**–**F**. The following are the scale bars: 500 µm for the dentate gyrus at low magnification, and 20 µm for all other images. Statistical significance: *p < 0.05, **p < 0.01, and ***p < 0.001 vs. saline; and #*p* < 0.05, ##*p* < 0.01, and ###*p* < 0.001 vs. Sco

**Fig. 4:**
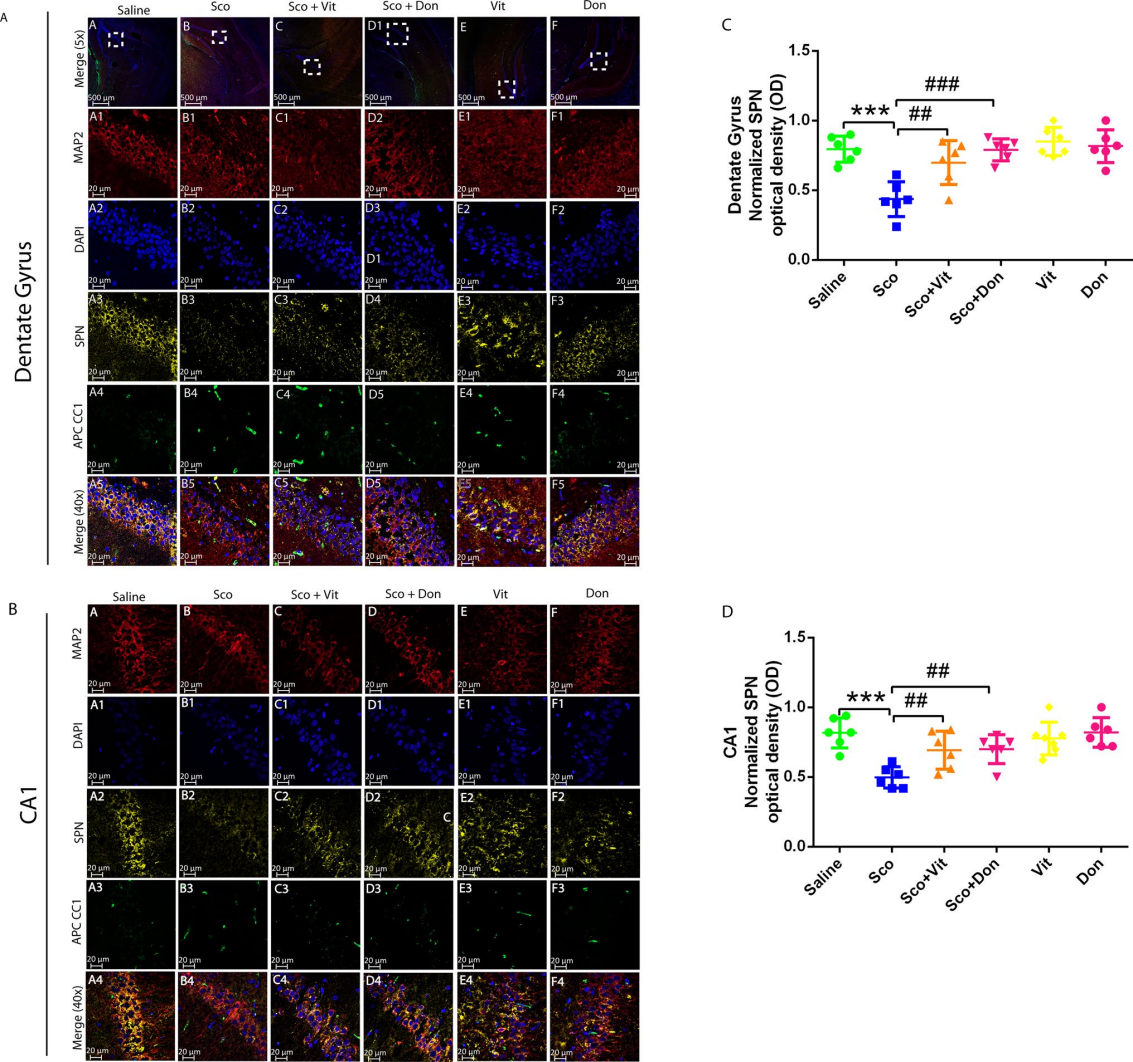
High-resolution confocal immunofluorescence images that demonstrate the expression patterns of microtubule-associated protein 2 (MAP2, red), synaptophysin (yellow), APC/CC1 (green), and DAPI (blue) in the hippocampal dentate gyrus and CA1 region across experimental groups. MAP2 staining delineates dendritic structures and neuronal soma, synaptophysin indicates presynaptic vesicles, CC1 marks mature oligodendrocytes, and DAPI represents nuclei. Exposure to Sco revealed a significant reduction in synaptophysin expression, which indicates a loss of presynaptic terminals. In contrast, the expressions of MAP2 and CC1 remained largely stable, thus suggesting that the dendritic architecture and oligodendrocyte populations were mostly maintained. Treatment with Vit (Sco + Vit) or Don (Sco + Don) resulted in the restoration of synaptophysin expression to levels comparable to the saline group, thereby indicating the preservation of presynaptic integrity. No notable changes were detected in MAP2 or APC/CC1 signals among the groups, thus suggesting that Vit and Don primarily provided protective effects at the synaptic level instead of causing substantial alterations in neuronal or myelin structures. The quantitative analyses of synaptophysin optical density (OD) values are presented in panels C and D, thus corroborating the histological observations of maintained synaptic integrity following treatments with Vit and Don. No differences were observed between the groups for MAP2, DAPI, and APC/CC1 protein expressions. Scale bars indicate a measurement of 500 µm for low magnification images and 20 µm for high-magnification images. Statistical significance: *p < 0.05, **p < 0.01, ***p < 0.001 vs. saline; and #*p* < 0.05, ##*p* < 0.01, ###*p* < 0.001 vs. Sco

The immunofluorescence evaluations of hippocampal slices demonstrated significant changes in glial activation and synaptic integrity after Sco administration. Sco treatment resulted in significant astrocytic reactivity and synaptic protein loss in both the dentate gyrus and CA1 regions, which were effectively reduced by Vit and Don treatments. GFAP immunoreactivity, indicative of astrocyte activation, was significantly increased in the Sco group relative to the saline group, thus suggesting the occurrence of reactive astrogliosis. In contrast, the expression of PSD95, an important postsynaptic marker, was significantly diminished, thereby indicating compromised synaptic stability. Both Vit and Don treatments significantly reduced GFAP expression and restored PSD95 levels, thus indicating decreased glial activation and preserved postsynaptic structure. CC1 immunoreactivity—which indicates the presence of mature oligodendrocytes—exhibited no significant differences across groups, thereby implying that oligodendrocyte populations were largely unaffected by Sco exposure or treatments. Further, MAP2 immunoreactivity and DAPI nuclear staining revealed no significant differences among groups, thus suggesting that Sco primarily influenced functional rather than structural neuronal parameters. In contrast, synaptophysin expression—a marker for presynaptic vesicles—was significantly diminished in the Sco group and was restored by both Vit and Don, thus indicating protective effects on presynaptic terminals. Quantitative optical density analyses demonstrated that Sco significantly elevated GFAP levels while reducing PSD95 and synaptophysin levels in the dentate gyrus; these changes were notably reversed by treatments with Vit and Don (Figs. 3 and 4). These findings suggest that Vit and Don provide neuroprotective and synaptoprotective effects by reducing astrocytic activation and maintaining the integrity of both pre- and postsynaptic structures in a Sco-induced cognitive impairment model.

#### Results of the Western Blot Analysis

##### Nrf2/HO-1 Signaling Pathway

**Fig. 5:**
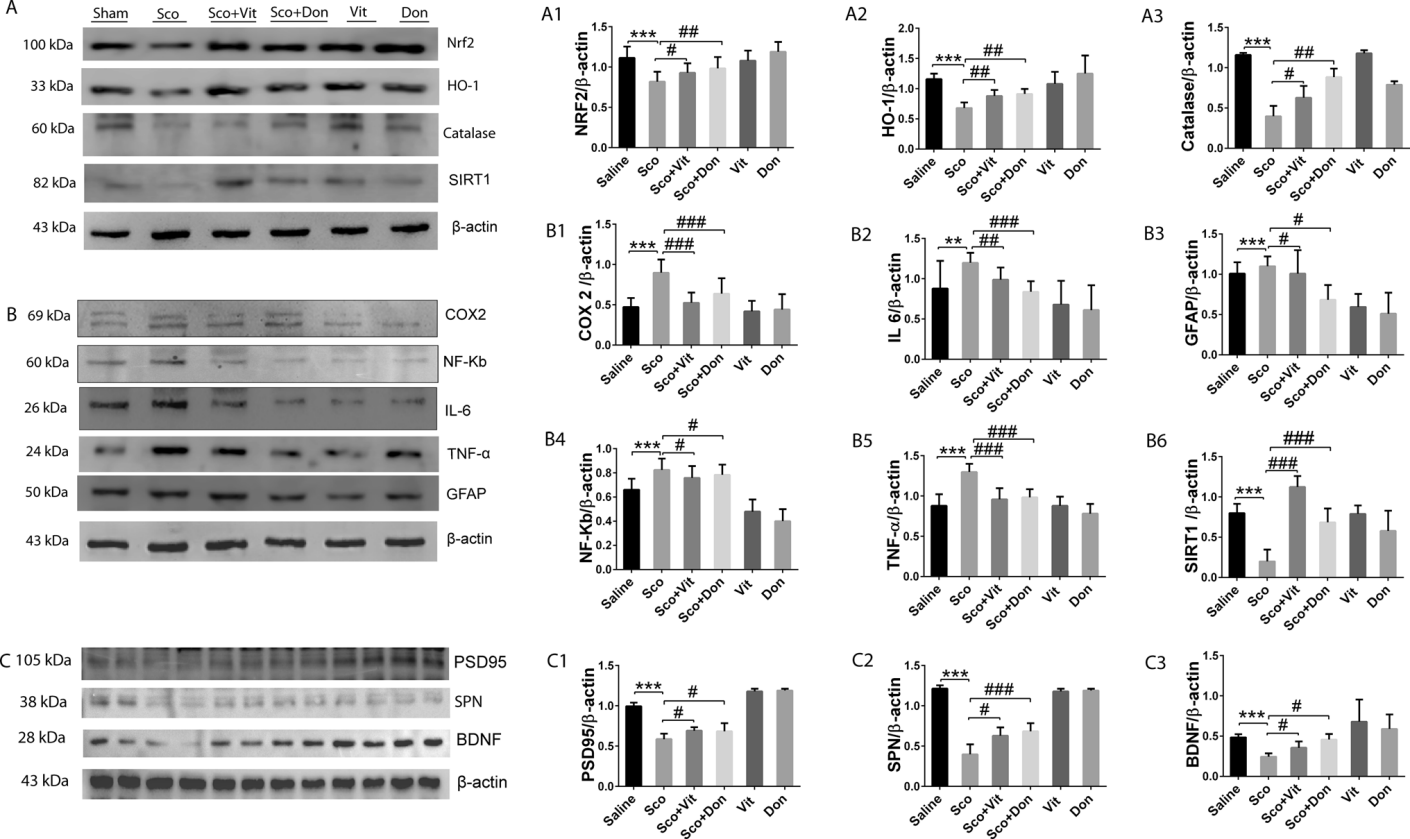
Western blot analysis of hippocampal protein expression among experimental groups. Representative blots displaying β-actin (~43 kDa) as a loading control for all studied proteins. Representative bands of Nrf2 (~100 kDa), HO-1 (~33 kDa), NF-κB p65 (~60 kDa), IL-6 (~26 kDa), GFAP (~50 kDa), Catalase (~60 kDa), COX-2 (~69 kDa), SIRT1 (~82 kDa), TNF-α (~24 kDa), PSD95 (~105 kDa), Synaptophysin (SPN, ~38 kDa), and BDNF (~28 kDa) are displayed. ***p < 0.001 vs. Saline group. ###*p* < 0.001, ##*p* < 0.01 compared to Sco. The experiments were performed twice

Western blot analysis demonstrated the effect of Sco and co-treatments on key hippocampal proteins, including SIRT1, Nrf2, and HO-1. In Fig. 5, panel A shows that Sco administration led to a significant reduction in Nrf2 protein levels compared to the saline group (*p* < 0.001). SIRT1 protein expression also exhibited a similar decreasing trend in the Sco group. Co-treatment with vitexin (Sco + Vit) or donepezil (Sco + Don) reinstated Nrf2 expression to levels comparable to the saline group (*p* < 0.05 and *p* < 0.01, respectively). In addition, HO-1 expression was significantly reduced in the Sco group (*p* < 0.001) but was notably restored after treatment with Sco + Vit or Sco + Don (*p* < 0.01) (Fig. 5).Catalase expression, an indicator of antioxidant defense, was significantly reduced in the Sco group. Treatment with vitexin (Sco + Vit) or donepezil (Sco + Don) significantly increased catalase expression compared to the Sco group ((p<0.05, p<0.01).

##### Anti-inflammatory Effect of Vit

Sco significantly increased the levels of proteins associated with inflammation. COX-2 expression significantly increased in the Sco group (*p* < 0.001) and was significantly reduced by Sco + Vit and Sco + Don (*p* < 0.001, *p* < 0.001). The expression of NF-κB p65 was significantly elevated by Sco (*p* < 0.05), with the combination of Sco and Don demonstrating a similarly suppressive effect compared to Sco and Vit (*p* < 0.05). Further, pro-inflammatory cytokines IL-6 and TNF-α were significantly elevated after Sco administration (*p* < 0.01, *p* < 0.001, respectively). The elevated TNF-α protein expression was significantly reduced in both treatment groups (*p* < 0.001) (Fig. 5B). The expression of IL-6 approached control levels in the Sco + Vit and Sco + Don groups (*p* < 0.01, *p* < 0.001), with Sco + Don exhibiting greater effectiveness (Fig. 5). GFAP expression was significantly increased in the hippocampus of Sco-treated rats relative to the saline group (*p* < 0.001). Co-treatment with Vit (Sco + Vit) or Donl (Sco + Don) substantially reduced the elevation in GFAP expression (*p* < 0.05). Notably, neither Vit nor Don alone affected GFAP levels in comparison to the saline group, indicating that their modulatory effects were exclusive to Scoe-induced glial reactivity. These findings indicate that Vit and Don effectively mitigate Sco-induced oxidative stress and neuroinflammation by restoring Nrf2/HO-1 signaling and inhibiting COX-2/NF-κB-mediated inflammatory pathways.

##### Alterations in Synaptic Protein Expression in the Hippocampus

In addition to oxidative and inflammatory damage, disruptions in synaptic integrity are key contributors to Sco-induced cognitive impairment. To evaluate synaptic plasticity, the expression levels of PSD95 and synaptophysin—established markers of postsynaptic density and presynaptic terminals, respectively—were analyzed by Western blot in hippocampal tissue. As shown in Fig. 5C, Sco administration significantly reduced the expression of both PSD95 (*p* < 0.05) and synaptophysin (*p* < 0.001) compared to the saline group, indicating impaired synaptic integrity. Treatment with Vit (Sco + Vit) or Don (Sco + Don) markedly restored PSD95 and synaptophysin levels relative to the Sco group (*p* < 0.05, *p* < 0.01), suggesting a protective effect on synaptic architecture. Neither Vit nor Don alone significantly altered baseline synaptic protein expression compared to the saline group. Similarly, BDNF expression was significantly decreased in the Sco group (*p* < 0.001), while both treatments effectively restored BDNF levels, with Sco + Don showing near-complete normalization (*p* < 0.05, *p* < 0.01). No significant differences were observed between the Vit- or Don-only groups and the saline group. Overall, these findings indicate that Vit and Don preserve synaptic integrity and neurotrophic support under conditions of Sco-induced neurotoxicity.

## Discussion

The present study demonstrates that vitexin exerts robust neuroprotective and synaptoprotective effects in a Sco-induced rat model of cognitive deficits and anxiety-like behavior. Vitexin ameliorated spatial learning and memory deficits in the MWM, reduced anxiety-like behaviors in the EPM, and restored hippocampal cholinergic, oxidative/nitrosative imbalance, inflammatory, neurotrophic, and synaptic homeostasis to levels comparable to those achieved by the gold-standard cholinesterase inhibitor donepezil.

Sco, a nonselective muscarinic acetylcholine receptor antagonist, is extensively utilized to induce memory impairment by disrupting cholinergic neurotransmission and modeling AD-like pathology in rodent models [37].

Sco-treated rats demonstrated elevated AChE activity, which is consistent with the cholinergic dysfunction reported in other research [38]. Treatment with Vit suppressed AChE activity in this study, thus corroborating prior results from in vitro studies that demonstrate Vit’s dual inhibitory effect on acetylcholinesterase and butyrylcholinesterase [39, 40]. In our study, consistent with previous findings, it was demonstrated that Sco notably impaired spatial learning and memory in the MWM test [41, 42]. The administration of Sco also elicited anxiety-like behavioral alterations, which is in accordance with prior findings [41, 43, 44]. Treatment with Vit significantly reduced these deficits, thereby suggesting its potential cognitive-enhancing and anxiolytic properties.

Prior studies have demonstrated that oxidative stress is a key factor in Sco-induced cognitive dysfunction, primarily due to the overproduction of ROS and the weakening of the antioxidant defense capacity [45–47]. In this study, Sco significantly decreased TAS and concurrently elevated TOS, lipid peroxidation (MDA), and nitrosative stress markers such as peroxynitrite, nitric oxide, and nitric oxide synthase [10, 48–50]. Moreover, the expression of Nrf2 and HO-1 proteins was significantly diminished in Sco-treated rats, thereby confirming the suppression of endogenous antioxidant defense systems. Both Vit and Don treatments reinstated Nrf2 and HO-1 expression and restored TAS levels while markedly reducing TOS, MDA, NO, and NOS, thereby demonstrating their antioxidant and anti-nitrosative properties, which are in accordance with previous studies [51–53]. GPx4—a crucial antioxidant enzyme that neutralizes lipid hydroperoxides and inhibits ferroptosis—was significantly downregulated in the Sco-treated group, thus signifying a profound disturbance of cellular redox equilibrium [54, 55]. Vit treatment markedly restored GPx4 expression, thereby indicating that its neuroprotective effects are, at least partially, facilitated by the activation of endogenous antioxidant defense mechanisms. The results are consistent with previous studies that indicate that Vit increases antioxidant enzyme activity and diminishes lipid peroxidation in neurotoxicity and aging models [56, 57].

Cognitive and memory impairments are closely associated with neuroinflammatory processes. In our study, repeated Sco administration in rats significantly elevated the levels of pro-inflammatory mediators—including NF-κB, TNF-α, COX-2, and IL-6—which is consistent with prior findings [58]. NF-κB, a key transcription factor that regulates cytokine production and inflammatory signaling, is widely expressed in the nervous system, particularly within synaptic terminals. Indeed, a studie has shown that NF-κB is absent in synapses derived from p65-deficient neurons [59], highlighting the essential role of the p65 subunit in the proper localization and function of NF-κB in hippocampal synapses. Moreover, the deletion of p65 or suppression of neuronal NF-κB through the super-repressor IκB leads to the loss of neuroprotection and deficits in learning and memory [60], indicating that physiological neuronal NF-κB activity supports cognitive processes. However, under pathological conditions, NF-κB activation can shift toward glial cells, where it acts as an inducible factor that promotes the transcription of pro-inflammatory cytokines. In this context, the overactivation of NF-κB enhances microglial activation and upregulates inflammation-related genes, contributing to neuroinflammatory damage [48, 56]. This mechanism likely explains the elevated p65 signaling observed in the scopolamine model, which predominantly arises from glial rather than neuronal sources. Activated glial NF-κB p65 promotes the production of TNF-α, IL-6, and COX-2, thereby exacerbating oxidative stress and neuroinflammation. Conversely, NF-κB suppression in glial cells may mitigate these pathological processes, whereas its physiological activation in neurons supports memory enhancement. In our study, Vit significantly diminished NF-κB p65 expression, confirming its potent anti-inflammatory capacity. A notable negative connection was identified between hippocampal inflammatory cytokine levels and both the duration spent in the target quadrant and the latency to find the hidden platform, thereby suggesting that increased inflammation correlates with diminished spatial memory function. In alignment with Abdallah et al. findings, which indicated enhanced MWM performance and diminished TNF-α levels in ICV-STZ animals, our data reveal that Vit administration reduced escape latency concurrently with decreased hippocampus NF-κB and TNF-α expression [61]. Further, Sco elicited significant activation of astrocytes and microglia, as evidenced by increased GFAP expression in the hippocampus consistent with existing literature. GFAP is a cytoskeletal protein produced by astrocytes and serves as a recognized marker of astroglial activation and astrogliosis. Reactive astrogliosis, frequently induced by oxidative and inflammatory damage, is a defining characteristic of neuroinflammatory disorder and is closely associated with cognitive impairment [62–64]. Vit treatment decreased GFAP levels, thereby signifying notable anti-gliotic effects that maintain neuronal connectivity.

At the neurotrophic level, Sco significantly downregulated BDNF and GDNF, both of which are crucial for synaptic plasticity and neuronal survival [65–67]. The reduction reflects findings that indicate that cholinergic dysfunction diminishes the BDNF expression in the hippocampus [68]. Vit treatment reversed this alteration, upregulated BDNF and GDNF expression, and, thereby, enhanced neuronal survival and synaptic plasticity. These findings align with earlier studies that suggest that elevated levels of BDNF and GDNF enhance cognitive function and protect against neurodegeneration [69–71]. Moreover, Sco markedly decreased the expression of synaptic markers PSD95 and synaptophysin, thus indicating compromised synaptic integrity and plasticity. These modifications align with prior research that indicates that Sco impairs synapse structure and transmission, thus leading to deficits in learning and memory [8, 72–74]. Although some studies have reported that the expression of synaptic markers such as PSD95 does not change following Sco administration, we believe that this discrepancy may be attributed to factors such as differences in experimental models, protocols, Sco dose, route of administration, and duration of treatment [75–77]. The administration of Vit significantly reversed these protein-level changes, thereby indicating that Vit suppresses inflammatory cascades and preserves pre- and postsynaptic protein expression. The simultaneous restoration of PSD95 and synaptophysin levels indicates a significant synaptoprotective effect, thereby aligning with the observed behavioral improvements and immunofluorescent results in the CA1 and dentate gyrus regions. In our study, APC (CC1) and DAPI staining revealed no substantial differences among the groups. The findings indicate that Sco administration in our protocol did not affect the quantity of mature oligodendrocytes or the overall nuclear cell density, with the observed impairment being primarily at the functional or synaptic level [78].

Numerous studies have indicated that Vit, a glycosylated flavonoid, provides substantial neuroprotection by inhibiting Aβ25–35-induced cytotoxicity in PC12 and Neuro-2a cells [79, 80]. Moreover, Vit has demonstrated the ability to protect transgenic *C. elegans* models from Aβ-related proteotoxic stress and possesses significant inhibitory effects on critical Alzheimer’s-associated enzymes, such as AChE and BACE1 [39, 81]. These findings indicate that Vit provides neuroprotection by modulating inflammatory and synaptic signaling pathways, thereby emphasizing its potential as a therapeutic candidate for alleviating early molecular events associated with cognitive dysfunction and neuroinflammation.

Considering its wide range of biological activities, the therapeutic potential of Vit has been investigated in numerous in vivo and in vitro disease models; it has even been evaluated in clinical studies as part of nutraceutical formulations. A phase 4 clinical trial (NCT01647984) demonstrated that a nutraceutical combination containing Vit alleviated arrhythmic load in benign ventricular and supraventricular extrasystoles. This indicates that formulations that contain Vit are well tolerated in people. Despite Vit’s inclusion in human nutraceutical research (e.g., NCT04245761), to the best of our knowledge, no randomized, placebo-controlled trial has assessed Vit monotherapy for cognitive impairment. In addition, the findings related to humans indicate the safety and preliminary therapeutic effects of Vit-containing supplements; nevertheless, additional dedicated trials are necessary to evaluate their efficacy in neurodegenerative illnesses.

While this study has yielded promising findings, it is subject to several limitations. The Sco-induced model predominantly reflects acute cholinergic dysfunction associated with AD and may not sufficiently replicate the chronic and progressive features of the condition [78, 82]. Thus, the prolonged neuroprotective effectiveness of Vit could not be assessed in this acute experimental context. Moreover, a single dose of Vit (30 mg/kg) was administered, and the possible effects associated with other dosages were not evaluated. Therefore, a dose–response analysis would provide critical insights into the therapeutic window and optimal concentration of Vit. Further, the study focused on oxidative stress, neuroinflammation, and synaptic markers; however, it did not assess other clinical features of AD, such as amyloid beta buildup and Tau hyperphosphorylation. Future studies that include chronic neurodegenerative models and thorough pathological evaluations are necessary to adequately assess the translational potential of Vit. Furthermore, research investigating the possible synergistic effects of Vit combined with standard cholinesterase inhibitors, such as Don, could yield important insights into improving therapeutic outcomes in AD. From a molecular standpoint, our data suggest that Vit confers neuroprotection by modulating BDNF, GPx4, and NF-κB signaling; however, further mechanistic investigations are required to further substantiate this.

## Conclusion

This study concludes that Vitexin efficiently alleviates cognitive and behavioral deficits induced by Sco through its multifaceted neuroprotective mechanisms. Vit reestablished oxidative balance by stimulating intrinsic antioxidant mechanisms, mitigated hippocampal inflammation through the downregulation of NF-κB p65–mediated pathways, augmented neurotrophic support by increasing BDNF and GDNF expression, and preserved synaptic integrity by maintaining PSD95 and synaptophysin levels. These data indicate that Vit provides extensive protection against synaptic, oxidative, and inflammatory alterations associated with cholinergic dysfunction. Based on these findings, Vit appears to be a promising candidate for further investigation in the prevention or mitigation of memory impairment in neurodegenerative diseases, including AD. Additional research that employs chronic models and mechanistic validation is necessary to further reveal the translational potential of Vit.

## Supplementary Information

Below is the link to the electronic supplementary material.

ESM 1 (PDF 246 KB)

## Data Availability

No datasets were generated or analysed during the current study.
