# Supplementary figures and images for: Vitexin Protects Against Scopolamine-Induced Cognitive Impairment by Preserving Synaptic Integrity and Modulating Nrf2/HO-1 and NF-κB Signaling Pathways
Source: Mol Neurobiol. 2026 Jun 25;63(1):722. doi: 10.1007/s12035-026-05947-0 (PMC13303689; doi:10.1007/s12035-026-05947-0)

FULL IMMUNOBLOTS

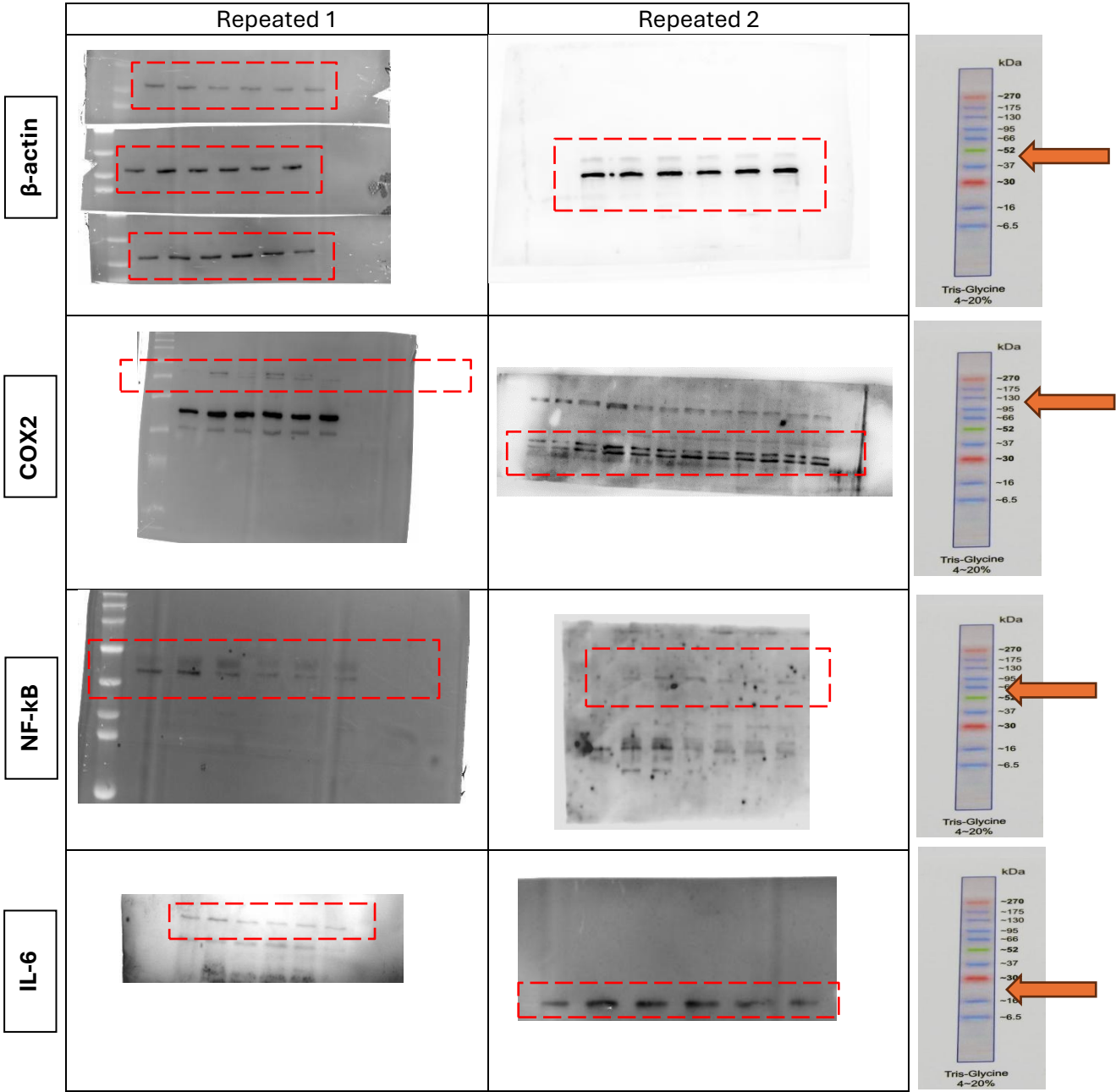

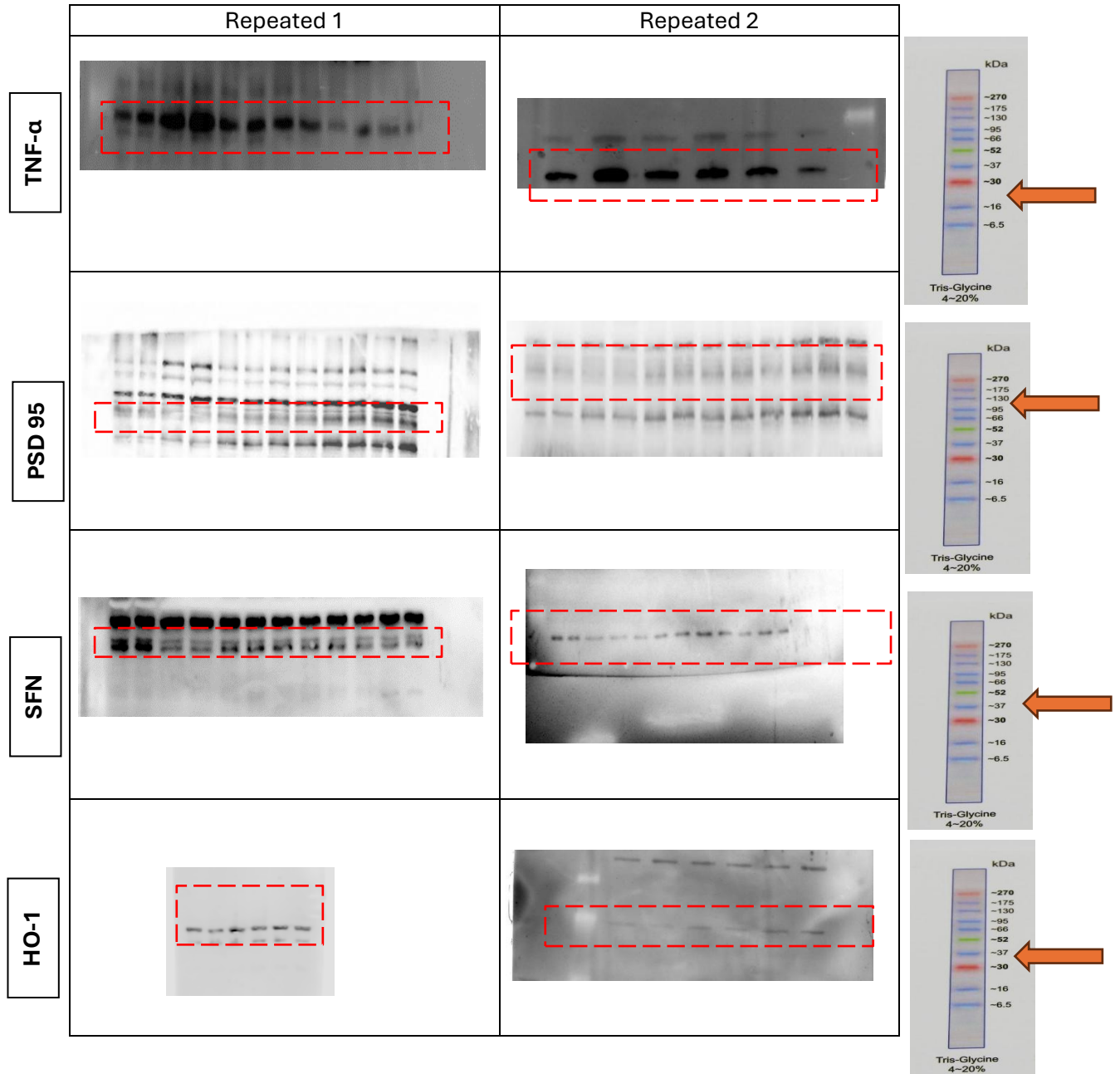

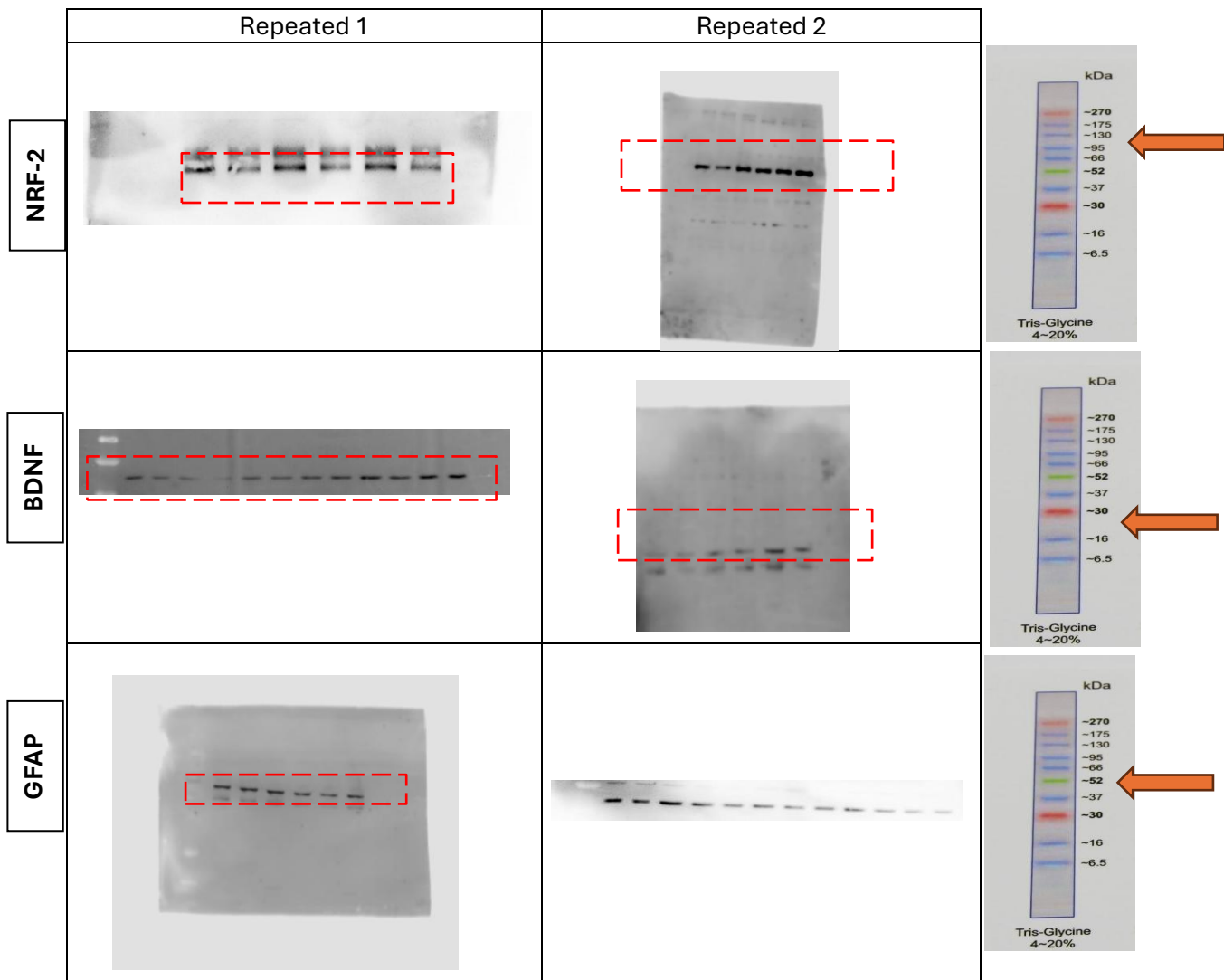

Supplement: Supplementary file 1 — (PDF 246 KB) [file 12035_2026_5947_MOESM1_ESM.pdf]
